# Nontuberculous Mycobacteria Isolation from Clinical and Environmental Samples in Iran: Twenty Years of Surveillance

**DOI:** 10.1155/2015/254285

**Published:** 2015-05-28

**Authors:** Ali Akbar Velayati, Parissa Farnia, Mohadese Mozafari, Mehdi Mirsaeidi

**Affiliations:** ^1^Mycobacteriology Research Centre, National Research Institute of Tuberculosis and Lung Disease (NRITLD), Shahid Beheshti University of Medical Sciences, P.O. Box 19575-154, Tehran 19569-44413, Iran; ^2^Section of Pulmonary, Critical Care, Sleep and Allergy Department of Medicine M/C 719, University of Illinois at Chicago, 840 S. Wood Street, Chicago, IL 60612-7323, USA

## Abstract

Nontuberculous mycobacteria (NTM) are opportunistic pathogens that are widely distributed in the environment. There is a lack of data on species distribution of these organisms from Iran. This study consists of a review of NTM articles published in Iran between the years 1992 and 2014. In this review, 20 articles and 14 case reports were identified. Among the 20 articles, 13 (65%) studies focused on NTM isolates from clinical specimens, 6 (30%) studies examined NTM isolates from environmental samples, and one (5%) article included both clinical and environmental isolates. *M. fortuitum* (229/997; 23%) was recorded as the most prevalent and rapid growing mycobacteria (RGM) species in both clinical (28%) and environmental (19%) isolated samples (*P* < 0.05). Among slow growing mycobacteria (SGM), *M. simiae* (103/494; 21%) demonstrated a higher frequency in clinical samples whereas in environmental samples it was *M. flavescens* (44/503; 9%). These data represent information from 14 provinces out of 31 provinces of Iran. No information is available in current published data on clinical or environmental NTM from the remaining 17 provinces in Iran. These results emphasize the potential importance of NTM as well as the underestimation of NTM frequency in Iran. NTM is an important clinical problem associated with significant morbidity and mortality in Iran. Continued research is needed from both clinical and environmental sources to help clinicians and researchers better understand and address NTM treatment and prevention.

## 1. Introduction

In 1996, the Working Group of the Bacteriology and Immunology Section of the International Union against Tuberculosis and Lung Disease contacted 50 laboratories in several countries, including Iran, in order to collect and analyze epidemiological data for nontuberculous mycobacteria (NTM) or mycobacteria other than tuberculosis (MOTT). At this time, the Iranian reference laboratory provided data from 98 patients (1980–1983), of which* M. fortuitum* and* M. kansasii *were identified as the most dominant NTM in clinical samples [1]. In the following years, many researchers attempted to determine the prevalence of NTM and its importance in Iran [2–4]. Unfortunately, these studies failed to capture a comprehensive measure of NTM in Iran. The majority of NTM in Iran consist of small samples or data confined to small geographical areas that cannot be generalized. As a result, no clear data on the epidemiology of NTM is available on the national scale.

Iran is an intermediate tuberculosis- (TB-) burden country where TB remains a major public health problem. The incidence of TB in Iran is 21 per 100,000 people.

The significant number of multi-drug resistant (MDR), extensive drug resistant (XDR), and totally drug resistant (TDR) tuberculosis underline the possibility of NTM infection among tuberculosis suspected cases [5, 6]. In most cases, patients with positive sputum smear microscopy are treated with first line pulmonary tuberculosis therapy. Clinical failures prompt the transfer of TB samples to central laboratories for further identification of isolates and in case of NTM infection. Therefore, the diagnosis and treatment of resistant TB begin with considerable delay [7]. So far, limited investigation on NTM infections is reported from TB endemic countries with limited laboratory resources. Instead, NTM infection is more documented in developed countries including geographical variability [8–10]. For example,* Mycobacterium avium* complex (MAC) followed by* M. gordonae *and* M. xenopi *is considered the most predominant NTM in the United States and Europe [11, 12]. Given the complex treatment challenges particularly in low resource countries, understanding geographical diversity of NTM within the country is particularly important. With this background, we aim to retrospectively analyze and compare the NTM data published in the last 20 years. In addition, we evaluated the long-term trends of NTM isolation from clinical and environmental specimens.

## 2. Methods

A literature search was performed in PubMed, Scopus, SID and Google Scholar, Embase, and the Cochrane Library on nontuberculous mycobacteria in Iran. The search keywords were “atypical Mycobacteria,” “nontuberculous mycobacteria,” and “Iran,” Original articles, case reports, and reviews published on nontuberculous mycobacteria in Iran in peer-reviewed journals including Persian and English journals were considered [2–4, 13–40]. Congress abstracts were excluded. The following data were abstracted for the purpose of review: the name of the city, research methods, and individual NTM species as well as sample source. The statistical significance of observed trends of NTM in the last 20 years was tested using Poisson log-linear regression. All analyses were performed using the statistical software packages SPSS version 21 (IBM SPSS, Inc., Chicago, IL).

## 3. Results

**Figure 1:**
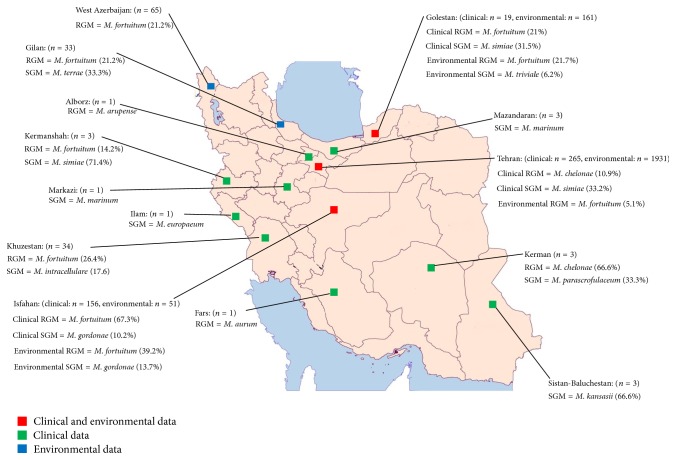
The provinces of Iran which work on NTM in clinical or environmental samples. The most prevalent SGM and RGM were noted.

Twenty original articles about NTM isolates were identified. The selected articles were published from 1992 to 2014. The majority of these articles (13/20; 65%) included data from clinical samples, six studies outlined the frequency of NTM in the environment, and a single (5%) article studied both clinical and environmental NTM (Figure 1). The geographical setting of these studies was Tehran in 6 articles (30%), Isfahan in 6 (30%), Khuzestan in 2 (10%), and Golestan in 2 (10%). The remaining articles included provinces such as Sistan and Baluchestan (1/20; 5%), Kerman (1/20; 5%), West Azerbaijan (1/20; 5%), and Gilan (1/20; 5%) (Table 1).

Among 14 case report articles from different cities of Iran, 4 were reported from Tehran (28.5%), 3 from Isfahan (21.4%), 2 from Sari (14.2%), and one from other cities including Shiraz (7.1%), Khomein (7.1%), Babol (7.1%), Ilam (7.1%), and Karaj (7.1%) (Table 2).

The majority of NTM species with known sources were isolated from respiratory specimens including sputum (134/494; 27.1%), bronchoalveolar lavage (51/494; 10.3%), bronchial washing (7/494; 1.4%), pleural samples (6/494; 1.2%), and lung tissue biopsy (5/494; 1%). Extrapulmonary samples were collected from urine (9/494; 1.8%), abscess (6/494; 1.2%), lymph node biopsy (4/494; 0.8%), gastric lavage (2/494; 0.4%), vaginal discharge (2/494; 0.4%), CSF (1/494; 0.2%), dermal lesion (3/494; 0.6%), subcutaneous nodule in hand or finger (4/494; 0.8%), and corneal biopsy (1/494; 0.2%). In a considerable number of reports (259/494; 52.4%), the sources of isolation were not documented.

As shown in Table 1, the primary method of NTM detection was based on culture using Löwenstein-Jensen media. Identification was performed by conventional methods in 38% (8/20) and molecular methods in 15% (3/20) of articles. In 9 (45%) studies, both molecular and conventional methods were applied.

### 3.1. NTM in Clinical Samples

The geographic locations of samples were mainly Tehran (261/480; 54.3%), Isfahan (153/480; 31.8%), Khuzestan (34/480; 7.1%), Golestan (19/480; 3.9%), Kermanshah (7/480; 1.4%), Kerman (3/480; 0.6%), and Sistan-Baluchestan (3/480; 0.6%). From 13 studies using clinical samples, 480 NTM species were isolated. Of these isolates, 269 (56%) were grouped as SGM and 211 (43.9%) as RGM. The most prevalent RGM in clinical samples was* M. fortuitum *(136/480; 28.3%) in all locations (Isfahan 105/153, 68.6%, Khuzestan 9/34, 26.4%, and Golestan 4/19, 21%) except for Tehran. The prevalence of RGM was* M. chelonae *(29/261; 11.1%) in Tehran. Among SGM species,* M. simiae *(103/480; 21.4%) showed the highest rate. Geographical distribution of SGM in clinical samples was* M. simiae* in Tehran (88/261; 33.7%) and Golestan (6/19; 31.5%),* M. gordonae* (16/153; 10.4%) in Isfahan, and* M. intracellulare *(6/34; 17.6%) in Khuzestan (Table 3).

In the case report articles,* M. marinum* (4/14; 28.5%) had higher detection rate, most frequently isolated from nodules or lesions of the hand (Table 2).

### 3.2. NTM in Environmental Samples

Data regarding environmental distribution of NTM were primarily from Tehran (193/503; 38.3%), Isfahan (51/503; 10.1%), Golestan (161/503; 32%), West Azerbaijan (65/503; 12.9%), and Gilan (33/503; 6.5%). In total, 503 NTM from environmental samples were isolated, which included 221 (43.9%) SGM and 282 (56%) RGM. Among RGM species,* M. fortuitum *(93/503; 18.4%) showed higher frequency in environmental samples in different locations including Golestan (35/161; 21.7%), West Azerbaijan (21/65; 32.3%), Isfahan (20/51; 39.2%), Tehran (10/193; 5.1%), and Gilan (7/33; 21.2%) (Table 4). Regardless of geographical locations, the frequency of* M. fortuitum* was high in both water (195/503; 38.7%) and soil (308/503; 61.2%) samples. SGM frequencies varied in different locations; in Gilan* M. terrae* was 11/33, 33.3%, in Golestan* M. triviale* was 10/161, 6.2%, and in Isfahan* M. gordonae* was 7/51, 13.7.

### 3.3. Trends of NTM

**Figure 2:**
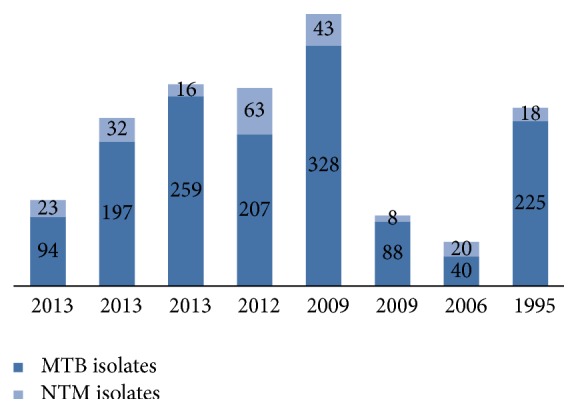
Number of NTM and MTB isolates in 8 studies.

As shown in Figure 2, the frequency of NTM among pulmonary TB cases was studied in only 8 studies. In 1995, 18 (8%) of 225 respiratory samples were recorded as NTM as compared to 2013 when 55 (18%) were recorded as NTM from 291 samples. This trend shows a significant increase in NTM detection rates during the study period (*P* < 0.05).

## 4. Discussion

To the best of our knowledge this is the first study in which trends in clinical and environmental NTM species have been investigated over the past twenty years. Overall, from 34 published reports (original and case reports), 997 NTM strains were identified (494 isolated from clinical samples and 503 from environmental samples). The majority of clinical (86.2%) and environmental (65.2%) NTM species were isolated from Tehran (*n* = 261) and Isfahan (*n* = 53), respectively. These data are incomplete considering that Iran consists of 31 provinces. Here, we showed that NTM was isolated only from 14 (46%) provinces during the past years (Figure 1). In the remaining 17 provinces, there is no data available on prevalence of environmental and clinical NTM. These numbers suggest that NTM is a neglected disease in Iran, which is likely true for other neighboring countries in the region, where the incidence of TB is higher, such as Afghanistan, Iraq, and Pakistan [41–48]. In Pakistan, three clinical NTM reports were published in 1984, 2011, and 2013 with total sample size of 4, 62, and 104 subjects, respectively [43, 44].* M. fortuitum* was identified as the most prevalent NTM in Pakistan (13.5%). In Iraq, few studies recently reported the frequency of NTM in dairy products and environmental samples such as milk powder and fresh milk, drinking water, and fecal samples from horses [46–48]. The most prevalent NTM species in Iraq was* M. chelonae *(18.2%) [46, 48].

In this study we also aimed to identify NTM distribution and trends within Iran. Variable techniques were used in different laboratories. From 1992 to 2006, most laboratories used traditional methods for identification of NTM in both clinical and environmental samples. However, from 2009 to 2014, advances in laboratory technique allowed combinations of traditional and molecular methods to be used (Table 1) resulting in the detection of more species of NTM in clinical and environmental samples. Reports from high-tech laboratories proposed the use of commercial line probe assay supplemented with sequencing for identification [49, 50]. Ideally, the use of the commercial method may support standardization and it facilitates the comparison of results within different settings. Our data demonstrate PRA (PCR restriction analysis) methods with either* hsp65* or* 16s-23s rRNA*,* rpoB* genes as the optional molecular test [18, 51, 52]. This highlights the need for standardized methods and guidelines for NTM identification in Iran.

We also showed that the majority of NTM were collected from respiratory samples. The results underline the importance of identifying NTM from suspected pulmonary TB patients. Molecular and phenotypic identification revealed a geographical distribution of NTM in Iran. From 494 clinical NTM isolates, 28.3% and 21.4% were recorded to be* M. fortuitum* and* M. simiae*, respectively. Analyzing the previous studies [15–19] showed geographical differences for* M. fortuitum *distribution, where Isfahan had the highest prevalence of* M. fortuitum* (105/153; 68.6%), while in Tehran the prevalence rate was less than 10% (17/261; 6.5%) (*P* > 0.05). These results make the analysis a bit difficult, as we are not sure if the report is a laboratory cross contamination or if it is* M. fortuitum *endemicity in some parts of Iran.

The results from environmental samples also showed the high frequency of* M. fortuitum* (93/282; 32.4%) followed by* M. chelonae *(38/282; 13.4%) in water and soil samples. This suggests the possible risk of* M. fortuitum* transmission from nature to human. Among SGM species,* M. simiae* is identified as the dominant NTM in Tehran (88/261; 33.7%) and Golestan provinces (6/19; 31.5%) [20, 53, 54]. In three other regional settings (Isfahan, Sistan-Baluchestan, and Kerman),* M. simiae* was not isolated. The clinical importance of* M. simiae* in various geographical regions of Asia, including Turkey and Japan, has been already documented [55, 56]. The frequency of* M. simiae* was reported to be from 1.5% to 10% across studies [53, 54]. For environmental SGM, the frequency of* M. flavescens *(44/503; 9%),* M. thermoresistibile* (24/503; 5%), and* M. terrae *(21/503; 5%) was higher than other species (Table 4). In contrast to RGM group, the distribution and frequency of slow growing mycobacteria in clinical and environmental samples were different.

The current study found a considerable number of environmental NTM (157/503; 31.2%) that remained unidentifiable (Table 3). This highlights the importance of the implementation of new techniques in order to improve NTM identification. At present, 8 regional and one national reference TB laboratories are functioning in Iran. Recently, due to global fund, they have been equipped with molecular diagnostic testing capabilities. As a result, it is our expectation that NTM detection will increase within the next few years.

Drug susceptibility tests (DST) for NTM were not performed in the majority of published studies in Iran. In developed countries, a variety of susceptibility testing methods such as the *E*-test, TREK, and microbroth dilution are used to carry out DST [57]. Given the well-described resistance patterns emerging in developed countries with low incidence of NTM, susceptibility testing is a particularly important clinical tool for countries such as Iran.

In conclusions, the trends of isolation and identification of NTM have been increased in Iran in the last 20 years. This increasing trend is attributable to the implementation of enhanced molecular techniques that have improved the detection coupled with the enhanced awareness of NTM in the clinical setting. However, further research is needed to address this important public health threat including enhancing the epidemiology of NTM throughout Iran, standardizing laboratory techniques for detection and drug susceptibility testing, and improving clinicians knowledge on NTM diagnosis and treatment in Iran.

## Figures and Tables

**Table 1:** Detection and identification procedures for nontuberculous mycobacteria (NTM) in different laboratories.

Region	Laboratory/year of publication	Number of isolated NTM	Detection	Identification
	Mycobacteriology Research Center (MRC)-NRIT LD-2014 [51]	Clinical isolates: 124	Smear microscopy and culture on solid media (Löwenstein-Jensen)	Amplification of *IS6110* and PCR-RFLP for *hsp65 *
	Mycobacteriology Research Center (MRC)-NRIT LD-2013 [52]	Environmental isolates (RGM): 36; total: 193	Smear microscopy and culture on solid media (Löwenstein-Jensen)	Amplification of *IS6110* and PCR RFLP of *hsp65* and *16 s–23 s rRNA *
Tehran	Department of Mycobacteriology, Pasteur Institute of Iran-2013 [2]	Clinical isolates: 32	Smear microscopy and culture on solid media (Löwenstein-Jensen)	Growth characteristics and pigmentation, biochemical properties, and PCR based on *hsp65 *
	Masoud Laboratory, TB Reference Centre of Ahvaz and Kermanshah, 2013 [54]	Clinical isolates: 23	Smear microscopy and culture on solid media (Löwenstein-Jensen)	Growth characteristics and pigmentation, biochemical properties, amplification, sequencing of *16S rRNA*, *rpoB*, *hsp65*, and *ITS *(Internal Transcribed Spacer)
	Masoud Laboratory-2012 [13]	Clinical isolates: 63	Smear microscopy and culture on solid media (Löwenstein-Jensen)	Growth characteristics and pigmentation, biochemical properties, amplification of *IS6110*, and PCR based on *hsp65 *
	Mycobacteriology Research Center (MRC)-NRIT LD-2009 [14]	Clinical isolates: 43	Smear microscopy and culture on solid media (Löwenstein-Jensen)	Growth characteristics and pigmentation, biochemical properties, and PCR-RFLP for hsp65

	Dept. of Microbiology, Isfahan University of Medical Sciences-2014 [15]	Clinical isolates: 34	Smear microscopy and culture on solid media (Löwenstein-Jensen)	Amplification and sequencing of *16SrRNA* and RFLP-PCR for *hsp65 *
	Dept. of Microbiology, Isfahan University of Medical Sciences-2013 [16]	Clinical isolates: 21	Smear microscopy and culture on solid media (Löwenstein-Jensen)	Growth characteristics and pigmentation and biochemical properties
Isfahan	Dept. of Microbiology, Isfahan University of Medical Sciences-2012 [17]	Environmental isolates: 21	Smear microscopy and culture on solid media (Löwenstein-Jensen)	Growth characteristics and pigmentation and biochemical properties
	Dept. of Microbiology, Isfahan University of Medical Sciences-2012 [18]	Environmental isolates: 22	Smear microscopy and culture on solid media (Löwenstein-Jensen)	Growth characteristics and pigmentation and biochemical properties
	Isfahan University of Medical Sciences-2011 [4]	Clinical isolates: 67	Smear microscopy and culture on solid media (Löwenstein-Jensen)	Growth characteristics and pigmentation, biochemical properties, amplification, sequencing of *16SrRNA*, and RFLP-PCR for *hsp65 *
	Isfahan University of Medical Sciences-2010 [19]	Clinical isolates: RGM:25	Smear microscopy and culture on solid media (Löwenstein-Jensen)	Growth characteristics and pigmentation, biochemical properties, genus and species specific PCR, and PCR based on *hsp65 *

Golestan	Health care centers of Golestan province-2013 [20]	Clinical isolates: 19	Smear microscopy and culture on solid media (Löwenstein-Jensen)	Growth characteristics and pigmentation, biochemical properties, amplification, and sequencing of *16S rRNA *
	Microbiology Laboratory of Urmia University of Medical sciences-2006 [21]	Environmental isolates: 161	Culture on solid media (Löwenstein-Jensen)	Growth characteristics and pigmentation and biochemical properties

Khuzestan	Tuberculosis reference laboratory, PHLS of Khuzestan province-2009 [22]	Clinical isolates: 8	Smear microscopy and culture on solid media (Löwenstein-Jensen)	Growth characteristics and pigmentation, biochemical properties, and PCR based on *hsp65 *
	Ahwaz University of Medical Sciences-1995 [23]	Clinical isolates: 18	Smear microscopy and culture on solid media (Löwenstein-Jensen)	Growth characteristics and pigmentation and biochemical properties

West Azerbaijan	Microbiology Laboratory of Urmia University of Medical Sciences-2010 [3]	Environmental isolates: 65	Smear microscopy and culture on solid media (Löwenstein-Jensen)	Growth characteristics and pigmentation and biochemical properties

Gilan	1992 [24]	Environmental isolates: 33	Culture on solid media (Löwenstein-Jensen)	Growth characteristics and pigmentation and biochemical properties

Sistan-Baluchestan	2006 [25]	Clinical: 3 NTM identified	Smear microscopy and culture on solid media (Löwenstein-Jensen)	Growth characteristics and pigmentation and biochemical properties

Kerman	2007 [26]	Clinical NTM: 3 NTM identified	Smear microscopy and culture on solid media (Löwenstein-Jensen)	Growth characteristics and pigmentation and biochemical properties

**Table 2:** Case report studies of Iran.

Species	Year	City	Infected organ
*Mycobacteriumbranderi* [27]	2014	Tehran	Bone marrow
*Mycobacterium marinum* [28]	2014	Sari	Papule-hand
*Mycobacterium iranicum* [29]	2013	Isfahan	Bronchoalveolar lavage/hand wound
*Mycobacterium arupense* [30]	2013	Karaj	Respiratory system and blood
*Mycobacterium chelonae* [31]	2013	Tehran	Sputum and cervical lymph node
*Mycobacterium aurum* [32]	2012	Shiraz	Corneal biopsy
*Mycobacterium europaeum* [33]	2012	Ilam	Sputum
*Mycobacterium marinum* [34]	2011	Khomein	Nodule on the dorsum of fourth finger
*Mycobacterium marinum* [35]	2011	Babol	Lesions and pustules of the right forearm
*Mycobacterium monacence* [36]	2012	Isfahan	Sputum
*Mycobacterium parascrofulaceum* [37]	2011	Isfahan	Vaginal discharge
*Mycobacterium lentiflavum* [38]	2010	Tehran	Sputum
*Mycobacterium marinum* [39]	2008	Sari	Nodules and bulls on the back of the right hand
*Mycobacterium thermoresistibile* [40]	2006	Tehran	Cervical lymph node

**Table 3:** Species distribution of clinical nontuberculous mycobacteria isolated in articles reported from Iran.

Species	Kermanshah (2013)	Kerman (2007)	Isfahan (2010–2014)	Khuzestan (1995–2009)	Tehran (2009–2014)	Sistan-Baluchestan (2006)	Golestan (2013)	Total
*M. fortuitum *	1		105	9	17		4	136
*M. simiae *	5			4	88		6	103
*M. kansasii *	1		14	2	38	2		57
*M. gordonae *			16	5	19		1	41
*M. chelonae *		2			29		1	32
*M. intracellulare *			2	6	11			19
*M. abscessus *					17			17
*M. scrofulaceum *				1	7			8
*M. avium *			1	3	1	1		6
*M. conceptionence *			3		2			5
*M. marinum *					1		3	4
*M. lentiflavum *			1		2		1	4
*M. thermoresistibile *			1		3			4
*M. szulgai *				2	1			3
*M. branderi *					3			3
*M. parascrofulaceum *		1	1		1			3
*M. gastri *					2		1	3
*M. malmoense *					3			3
*M. porcinum *			3					3
*M. phlei *			2		1			3
*M. massiliense *					3			3
*M. monacense *			1		1			2
*M. nonchromogenicum *					1		1	2
*M. senegalense *					2			2
*M. genavense *					1			1
*M. triviale *				1				1
*M. sherrissii *					1			1
*M. xenopi *				1				1
*M. montefiorense *					1			1
*M. triplex *					1			1
*M. arupense *					1			1
*M. nebraskense *					1			1
*M. flavescens *							1	1
*M. smegmatis *			1					1
*M. austroafricanum *			1					1
*M. elephantis *			1					1
*M. novocastrencse *					1			1
*M. aurum *					1			1

**Table 4:** Species distribution of environmental nontuberculous mycobacteria isolated in articles reported from Iran.

Species	Isfahan (2012-2013)	West Azerbaijan (2010)	Golestan (2006)	Gilan (1992)	Tehran (2013-2014)	Total
*M. fortuitum *	20	21	35	7	10	93
*M. flavescens *	1	10	33			44
*M. chelonae *	5	6	27			38
*M. thermoresistable *		4	20			24
*M. terrae *	2		8	11		21
*M. phlei *	1		14			15
*M. pregrinum *		11			3	14
*M. mucogenicum *	5	6			2	13
*M. gordonae *	7		4			11
*M. triviale *			10			10
*M. senegalense *					9	9
*M. xenopi *	1			7		8
*M. avium *	1			6		7
*M. abscessus *	2	3			1	6
*M. smegmatis *	4	2				6
*M. parafortuitum *					5	5
*M. fallax *	1		4			5
*M. conceptionence *	1				3	4
*M. gastri *			3			3
*M. kansasii *			1	2		3
*M. neoaurum *		2				2
*M. marinum *			2			2
*M. aurum *					1	1
*M. poriferae *					1	1
*M. obuense *					1	1
Unidentified SGM					157	157
